# Biochemical composition, transmission and diagnosis of SARS-CoV-2

**DOI:** 10.1042/BSR20211238

**Published:** 2021-08-06

**Authors:** Rajesh Ahirwar, Sonu Gandhi, Komal Komal, Geeta Dhaniya, Prem Prakash Tripathi, Vyas Madhavrao Shingatgeri, Krishan Kumar, Jai Gopal Sharma, Saroj Kumar

**Affiliations:** 1 Department of Environmental Biochemistry, ICMR-National Institute for Research in Environmental Health, Bhopal 462030, India; 2 Diagnostic Laboratory, DBT-National Institute of Animal Biotechnology, Hyderabad 500032, India; 3 School of Biosciences, Apeejay Stya University, Gurugram 122103, India; 4 Division of Endocrinology, CSIR-Central Drug Research Institute, Lucknow 226031, India; 5 Cell Biology and Physiology Division, CSIR-Indian Institute of Chemical Biology, Kolkata 700032, India; 6 Cell Biology and Physiology Division, IICB-Translational Research Unit of Excellence, Kolkata 700091, India; 7 Department of Chemistry, Motilal Nehru College, University of Delhi, South Campus, New Delhi 110021, India; 8 Department of Biotechnology, Delhi Technological University, Delhi 110042, India

**Keywords:** COVID- 19, ELISA, RT-PCR, SARS-CoV-2, Transmission

## Abstract

Coronavirus disease 2019 (COVID-19) is a life-threatening respiratory infection caused by severe acute respiratory syndrome virus (SARS-CoV-2), a novel human coronavirus. COVID-19 was declared a pandemic by World Health Organization in March 2020 for its continuous and rapid spread worldwide. Rapidly emerging COVID-19 epicenters and mutants of concerns have created mammoth chaos in healthcare sectors across the globe. With over 185 million infections and approximately 4 million deaths globally, COVID-19 continues its unchecked spread despite all mitigation measures. Until effective and affordable antiretroviral drugs are made available and the population at large is vaccinated, timely diagnosis of the infection and adoption of COVID-appropriate behavior remains major tool available to curtail the still escalating COVID-19 pandemic. This review provides an updated overview of various techniques of COVID-19 testing in human samples and also discusses, in brief, the biochemical composition and mode of transmission of the SARS-CoV-2. Technological advancement in various molecular, serological and immunological techniques including mainly the reverse-transcription polymerase chain reaction (RT-PCR), CRISPR, lateral flow assays (LFAs), and immunosensors are reviewed.

## Introduction

**Figure 1:**
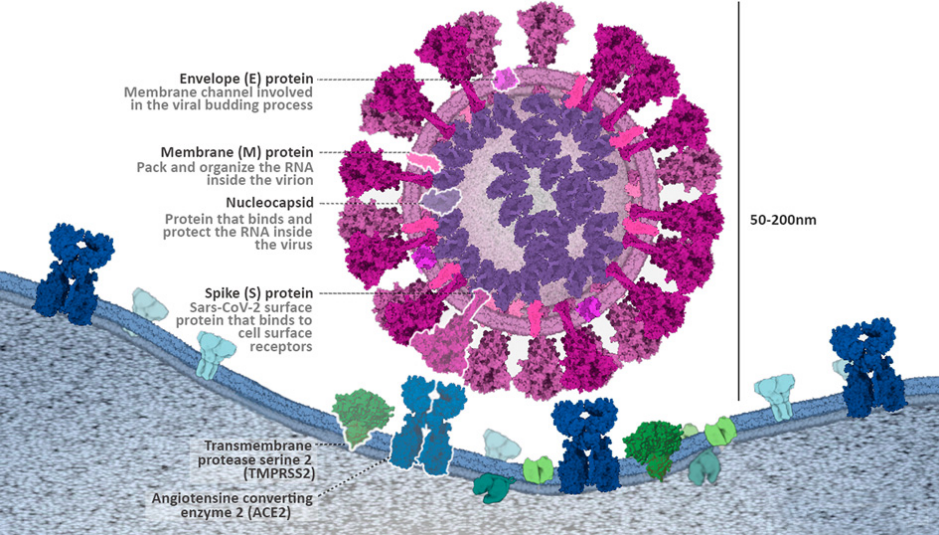
Structure of SARS-CoV-2 Schematic representation of the SARS-CoV-2, and its interactions via surface proteins with target cells. Image adapted from the educational portal of RCSB PDB (PDB-101). Image created by Marta Palma Rodríguez (Graduate Student, Hospital General Universitario de Valencia).

In the month of December 2019, a novel infectious disease named Coronavirus disease 2019 (COVID-19) was identified in the Wuhan city of Hubei province, China. Initial assessments on samples of a cluster of patients admitted with fever, cough and shortness of breath revealed pneumonia of unknown origin, later proved to be a viral infection by pathogen belonging to *Betacoronavirus* B lineage [1]. Based on genetic similarities to the genome of Middle East respiratory syndrome virus (MERS-CoV), severe acute respiratory syndrome virus (SARS-CoV) and bat coronavirus RaTG13, this novel virus was named SARS-CoV-2 [1–3]. Bats that have been considered the natural habitat for various coronaviruses including SARS-CoV-like and MERS-CoV-like viruses were predicted, based on the results of genome sequencing and evolutionary study, to be the suspected natural host in the origin of the SARS-CoV-2 [4–6]. There are different types of coronavirus that cause infections in human beings and SARS-CoV-2 is seventh in this series [2,7,8]. There are four different genera of coronavirus: α-CoV, β-CoV, γ-CoV, and δ-CoV. The α- and β-CoV cause infection in mammals, whereas γ- and δ-CoV infect the birds. Earlier, six CoVs were acknowledged as human-derived viruses, among them, HCoV-NL63 and α-CoVs HCoV-229E, and β-CoVs HCoV-HKU1 and HCoV-OC43 have low pathogenicity and cause minor respiratory symptoms, such as the common cold in humans [9]. SARS-CoV-2 on the other hand is more potent than SARS-CoV and MERS-CoV, and can lead to death due to pneumonia [10,11]. COVID-19 was shown exhibiting high viral shedding in the upper respiratory tract at an early stage of infection, resulting in a high proportion of transmission-competent individuals that are pre-symptomatic, asymptomatic, and mildly symptomatic [12]. For this, SARS-CoV-2 transmission is seen most commonly among people coming in close contact with patients or incubator carriers. Asymptomatic cases are another challenge for their ability to spread the virus without being producing noticeable symptoms in the infected individuals. It has been found that infection by SARS-CoV-2 could use a similar host protein named angiotensin-converting enzyme 2 (ACE2) as used by the SARS-CoV, to infect humans [1]. ACE2 is expressed by mnay tissues, for example, by the epithelial cells lining the nose, mouth, and lungs and acts as a major entry point for the COVID-19 virus. Recent findings indicate that SARS-CoV-2 use a cell surface complex that comprises a primary receptor called ACE2 and a serine protease named transmembrane protease serine 2 (TMPRSS2) co-localized on host cell surfaces to enhance the spike (S) protein-mediated entry of SARS-CoV-2 to host cell (Figure 1) [13]. Glycoproteins called S or spike proteins are viral envelope proteins comprising two active domains—the S1 domain that harbors receptor binding sites and also causes bacterial attachment to a cellular receptor, and the S2 domain that contains proteolytic cleavage sites to facilitate viral entry into target cells [13,14]. Once the ligand–receptor binding occurs, the TMPRSS2 cuts spike glycoprotein and controls the entry of viral envelopes into host cells through various synchronization changes [15]. The blockade of the ACE receptor can be controlled by inhibiting TMPRSS2 protease inhibition [16,17].

Accurate, affordable, and rapid testing holds key importance in timely diagnosis and isolation of infected individuals to curtail SARS-CoV-2 spread from both symptomatic and asymptomatic viral carriers [18]. Different types of COVID-19 tests, including mainly the reverse-transcription polymerase chain reaction (RT-PCR) for detection of viral RNA, lateral flow immunochromatographic strips for automated rapid antigen test, chest CT scan for analysis of associated clinical symptoms, and antibody test for analysis of SARS-CoV-2 antibodies have been used to detect the presence of SARS-CoV-2 or the body’s response to the infection [19–24]. Besides these, methods based on the use of electrochemical detection systems, isothermal nucleic acid amplification, nucleic acid microarrays, high-throughput sequencing, and serological and immunological assays based on colorimetry, fluorescence, and luminescence are also being developed to improve sensitivity, affordability, and diagnostic capacity for mass testing and assist in preventing future epidemics [25–27]. In this review, we summarize the current knowledge on biochemical composition, transmission modes, and diagnostic tools for the SARS-CoV-2. Notwithstanding the continuously evolving knowledge on this novel virus and measures of its testing and control, this review is intended to inform the audience of the causative agent of the ongoing pandemic and tools for detection of SARS-CoV-2 infection.

## Biochemical composition of SARS-CoV-2

SARS-CoV-2 is a single-stranded, positive-sense RNA virus, with structure similar to that of previously known SARS-CoV [28]. It contains four key building proteins: spike (S protein), membrane (M protein), envelope (E protein), and nucleocapsid (N protein) as shown in Figure 1.

Among the four binding proteins, coronavirus S protein is a transmembrane glycoprotein present on the viral surface and known to facilitate viral entry into target cells through the SARS-S/ACE2/TMPRSS2 route [16,29]. It is composed of an N-terminal S1 domain and C-terminal S2 domain, with three receptor-binding S1 heads sitting on top of a trimeric membrane fusion S2 stalk [30]. The flexible amino-terminal region of the spike protein (S1) has been shown to contain receptor-binding activity [31]. The highly stored S2 region consists of a transmembrane anchor, a palmitic acid acylation site [32] essential for membrane integration [33,34], and a coiled-coil fusion motor domain [35–40]. The S protein homotrimers are needed to attach to host receptors [41]. Proteolytic cleavage of SARS-CoV-2 spike promotes virus entry into the cell where it unpacks the viral RNA to replicate it and synthesize structural proteins required to assemble new viral particles and release them to infect other cells. The coronavirus E protein is the major complex and the smallest of the major building proteins [42]. It participates in the activation of pathogenesis, reunification, and discharge of the virus [43]. E proteins are produced by all recognized genome coronaviruses and originated at low levels in virion [44,45]. As highlighted by Kuo et al. [46], the E proteins appear to have three different functions that give to infection: controls the normal M–M interaction [47], disrupts the Golgi association in a way that produces large vesicles that are able to transmit virions [48–50], and interact with adhesive agents so that it can affect pathogenesis [51–56]. Similarly, coronavirus N proteins serve multiple functions, such as protects the viral genome, facilitates the interaction of protein M required through virion mating, and improves the effectiveness of viral transcription [57,58]. In addition, the N protein has a high immunogenic commotion and is extremely high during infection [59]. Therefore, protein N is a latent basis of diagnostic antigen for SARS-CoV-2 infection. Many N-protein-based diagnostic methods are designed to detect SARS-CoV [60–62]. In addition, different CoVs have specific building and support proteins, such as HE protein, 3a/b protein, and 4a/b protein [63]. Lastly, the M proteins are the most profuse viral protein present in the virion cell that provides a clear shape to the viral envelope [64]. It binds to the nucleocapsid and acts as a central controller of the COVID assembly [65]. M–M interaction picks up general scaffolding for the virus envelope. S-proteins and small amounts of E molecules are added to the M protein lattice in mature virions [66].

SARS-CoV-2 is genetically similar to the SARS-CoV. The results of the genomic picture comparison of the SARS-CoV-2 and SARS-CoV revealed the highest homology between the two nucleotide-type species. Furthermore, the genomes of these two species differ from each other in six regions. The first three distinct regions are sequential ORF1a/b coding sequences (448, 55, and 278 nt, respectively). The next two regions belong to several genetic sequences of gene S (315 and 80 nt, respectively) and the last diversity region is part of the genetic sequence of orf7b and orf8 genes (214 nt) [67]. The SARS-CoV-2 type spike shows much of the homology in bat-CoV, while the two genes 3a and 8b have homology in SARS-CoV.

Analysis of the protein similarity of SARS-CoV and SARS-CoV-2 suggested that most proteins are very homologous (95–100%). RdRp and 3CLpro protease share more than 95% sequence similarity although at genome level the two species share only 82% similarity [28,68–70]. In addition, both viruses share 76% consecutive similarities in their S proteins, the highly secreted receptor-binding domain (RBD), and the S protein domain [28,68–70]. Also, the PLpro sequence of SARS-CoV and SARS-CoV-2 shared 83% similarity with a large number of similar active sites [70]. All these pieces of evidence suggest the general evolutionary history of both species.

## Overview of transmission and interaction of SARS-CoV-2 with host

**Figure 2:**
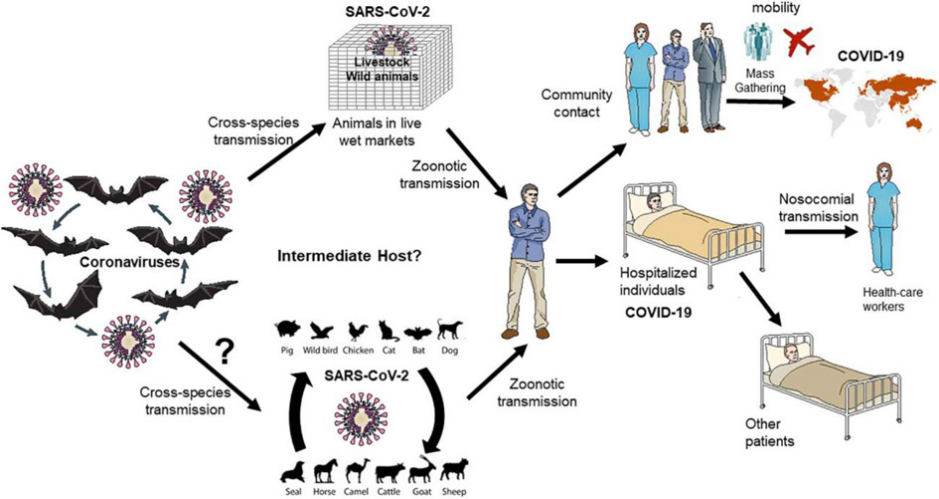
Transmission of epidemic zoonotic coronavirus Figure shows the transmission and interaction of coronavirus with host. Image adapted from [97] with permission from Elsevier under a Creative Commons CC-BY license.

SARS-CoV, MERS-CoV, and SARS-CoV-2 are RNA viruses belonging to the family Coronaviridae, the Orthocoronavirinae subfamily, β genus that causes the most respiratory infections in humans [2,12,71]. The genomes of SARS-CoV-2 are in 80% homology with SARS-CoV and 50% with MERS-CoV but only 96% of its coronavirus is isolated from large Yunnan bat [1]. In humans, coronavirus pathogenesis and infectivity are associated with a clear binding between the viral S protein, the glycoprotein found as a trimer on the exterior of the envelope lipid coating, and the protease receptor on a human cell part [72]. Respiratory drops are the main means of transmission; SARS-CoV-2 can be transmitted to a healthy person in case they come in contact with an infected person or one of his or her personal belongings, including clothing, door hinges etc. (Figure 2). People infected with SARS-CoV-2 commonly reported symptoms of fever, shortness of breath, cough, myalgia, fatigue, anosmia, ageusia, and normal or decreased leukocyte count [73]. Headaches, hemoptysis, abdominal pain, and diarrhea were also reported, though less frequently in COVID-19 patients [74]. It has been investigated that airborne transmission is also possible for SARS-CoV-2, but there is no definitive study on maternal infections (mother and child) [2,75–80]. Maintaining an appropriate distance among individuals, wearing eye protection, N95 respirators and surgical masks, and isolating the infected people are some of the Covid-appropriated behaviors found to control SARS-CoV-2 transmission [81]. Reports have also shown that, alike SARS-CoV, the half-life of aerosolized SARS-CoV-2 virus is approximately 1.2 h in the lab environment. Nevertheless, a recent study comparing the aerosol and surface (plastic, stainless steel, copper, and cardboard) stability of SARS-CoV-2 with SARS-CoV-1 found that the SARS-CoV-2 remained viable in aerosols for a prolonged time (e.g. <3 h), with a reduction in infectious titer from 10^3.5^ to 10^2.7^ TCID_50_ per liter of air. Also, the virus particles were found more stable (detected up to 72 h) on plastic and stainless steel than on copper and cardboard, although the virus titer was greatly reduced by 72 h [82]. According to available information, Morawska and Cao (2020) have suggested that small particles containing the virus can travel to indoor areas, covering distances up to 10 meters from extraction sources, thus facilitating aerosol transfer [83]. Likewise, Paules et al. (2020) pointed out that SARS-COV-2 air transmission is also possible without very long communication [84]. Both dynamic methods of assessment and calculation support these ideas. The infection is transmitted by large droplets produced during coughing and sneezing in patients with symptoms but can also occur in people without symptoms and before the onset of symptoms [85]. Patients can be contagious even after seroconversion [86]. A few studies found SARS-CoV-2 RNA detected in the upper respiratory specimens of the recovered patients for up to 12 weeks after symptom onset [87]. Also, COVID-19 has been found to affect more men than women [88–90]. The difference in mortality rate among males and females may result from presence of ACE2 encoding gene on the X-chromosome, differences in genomic distribution of so-called COVID-19 resistant alleles, and sex hormones (estrogen and testosterone) that have different immune functions that can affect differently the immunity or the severity of the disease [91,92]. According to current literature data, the mortality rate is 45-times higher in 30–39 year people, and 8700-times higher in 85+ age people when compared with 5–17 year age group [93]. It is opined that older adults have atypical presentation of symptoms that lead to a diagnostic and therapeutic delay which aggravates the prognosis of COVID-19 [94]. Also, higher complications have been seen in infected patients already suffering from diabetes, severe asthma, heart disease, obesity, hematological disease, recent cancer, kidney, liver, neurological or autoimmune environments [95]. It is also found that members of small communities such as blacks and southern Asians were at higher risk of contracting the disease [96].

## COVID-19 testing

COVID-19 is a highly infectious disease that transmits faster than other coronaviruses and causes fatal pneumonia in the infected individuals. Diagnostic tests for SARS-CoV-2 can be targeted to detect the virus or the immune response elicited in response to the viral infection. Multiple serological and immunological, molecular, and point-of-care tests are developed over the past year to assist in accurate, fast, and cost-effective diagnosis of SARS-CoV-2 infection.

### Sample for COVID-19 testing

**Figure 3:**
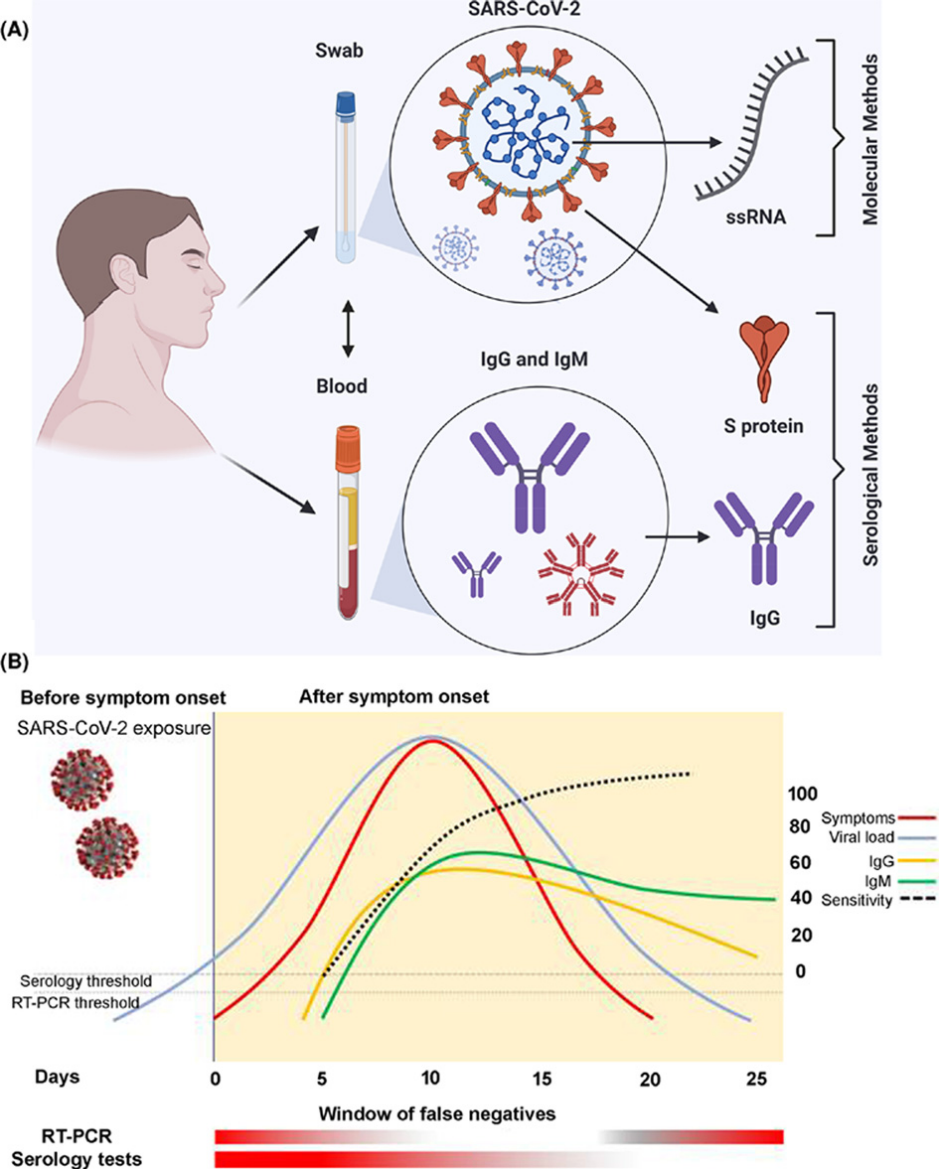
Samples and methods for testing COVID-19 Image (**A**) shows the two major categories of samples and corresponding methods for detection of COVID-19. Image (**B**) provide time relationship between viral load, symptoms, and positivity on sensitivity of diagnostic tests. After onset of symptoms (usually 5 days after exposure), the viral load could be below the threshold level for detection through RT-PCR (test may give false-negative results). The same pattern follows at the end of the disease when the patient is recovering. Seroconversion may usually be detectable between 5–7 and 14 days after the onset of symptoms. Thus, the serological tests are prone to give false-negative results in the first phase of the disease. Images adapted from [103] with permission from *PeerJ* under Creative Commons Attribution License, and [100] with permission from Elsevier under COVID-19 resource centre.

Method and site of specimen collection is very crucial for efficient and accurate detection of COVID-19. SARS-CoV-2 viral load in the respiratory tract is usually suggested to go in parallel with the viral dynamics in body fluids and tissues. While the nasopharyngeal swabs are the widely used specimen for to confirm SARS-CoV-2 infections through RT-PCR, specimen collection from other sites, such as the upper respiratory tract (containing the throat, deep throat saliva), the lower respiratory tract (containing sputum and bronchoalveolar lavage fluid), nasopharynx, feces, and blood has also been reported in detection of the SARS-CoV-2 infection [98,99]. For example, a study analyzed 1070 specimens from 205 COVID-19 patients (mean age: 44 years; range: 5–67 years; 68% male) and found highest positive rate in bronchoalveolar lavage fluid specimens, followed by sputum, nasal swabs, fibrobronchoscope brush biopsy, pharyngeal swabs, feces, and blood [98]. At present, collecting and testing upper respiratory specimen (nasopharyngeal) is recommended as first choice, if not possible, can be alternated with an oropharyngeal specimen, a nasal mid-turbinate specimen, an anterior nares specimen or a nasopharyngeal wash/aspirate or nasal aspirate for swab-based SARS-CoV-2 testing [100]. Although nasopharyngeal or throat swabs are preferred sites for sampling due to high positive rate, the sampling process may cause pain, coughing or sneezing in patients, creating a health hazard for medical personnel. Also, sputum (induced) have been suggested an effective specimen to detect SARS-CoV-2 RNA in recovering patients [101,102]. Notwithstanding the low reliability/sensitivity, detection of SARS-CoV-2 infection through serological/immunological tests on patients’ blood samples is another mostly used method after the molecular tests on swap-based specimen (Figure 3).

### Serological and immunological tests for SARS-CoV-2

Serological and immunological assays are used to monitor the progress of disease stages and to get information on past infection and immunity. These tests assess the presence of antibodies (serological tests) such as immunoglobulin M (IgM) and immunoglobulin G (IgG) antibodies or viral proteins (immunological tests) in the serum, plasma, saliva, sputum, or other biological fluids of patients. The methodologies used for determination of COVID-19 antigens and antibodies include enzyme-linked immunosorbent assay (ELISA), immunochromatographic lateral flow assay (LFA) (or rapid diagnostic test; RDT), neutralization bioassay, biosensors, and rapid antigen test are explained below.

### ELISA

**Figure 4:**
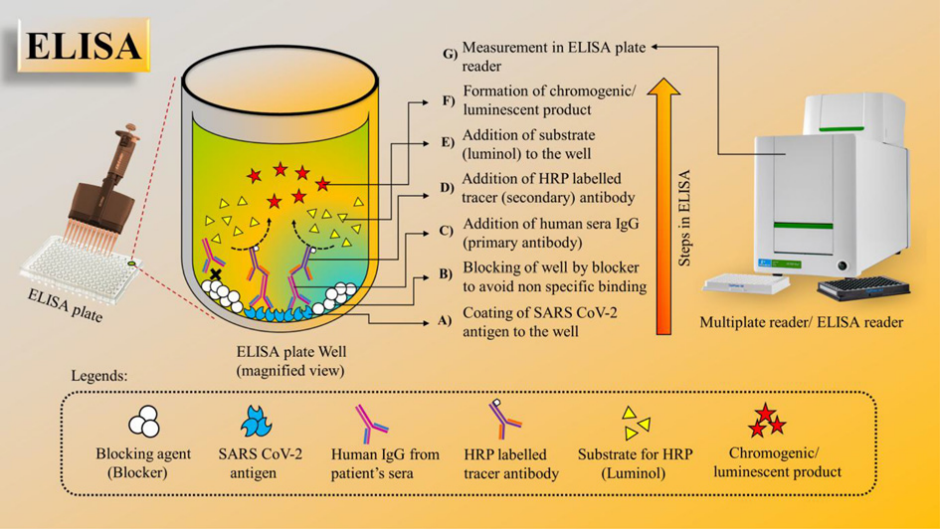
ELISA for the detection of SARS-CoV-2 antibodies in clinical serum samples (A,B) Coating of 96-well ELISA plate by optimized concentration of SARS-CoV-2 antigen followed by blocking. (C,D) Clinical serum samples added to the plate and allowed to incubate followed by addition of horseradish peroxidase (HRP)-labeled secondary antibody (Ab). (E) Addition of substrate. (F) Formation of a chromogenic product. (G) The intensity of the chromogenic product formed measured using an ELISA plate reader and further analysis of the concentration of SARS-CoV-2 Abs present in the clini cal serum samples.

ELISA is a plate-based technique designed to detect and measure substances such as peptides, proteins, antibodies, and hormones. The test can be performed in a normal 96-well microplate in a variety of formats such as direct, indirect, competitive, or sandwich with an effective assay time of usually 1−5 h. For example, a direct ELISA for SARS-CoV-2 detection would require coating the wells of microplates with SARS-CoV-2 antigen, followed by blocking of wells and addition of patients sera. If the patient has a recent infection of SARS-CoV-2, the antiviral antibodies (e.g., IgG) present in patient sera will be specially bound to coated antigen and the antibody–protein complex can be detected with an additional tracer antibody (linked to enzyme) to produce colorimetric or fluorescent-based readout. In general, ELISA is fast, has the ability to test multiple samples, and is adaptable to scale up [27]. The immunoassay-based COVID-19 detection methods are discussed in the detail in multiple previous studies [104–106]. The procedure of ELISA for the detection of SARS-CoV-2 antibodies in clinical serum samples is schematically represented in Figure 4.

### Lateral flow assay

Lateral flow assays, also known as lateral flow immunochromatographic assays, are simple immunoassay based on diagnostic devices used to detect the presence or absence of a target analyte (e.g. antibodies) in biological or other samples. These are typically qualitative (positive or negative) RDTs used for point-of-care testing (POCT). One type of RDT detects the presence of viral proteins (antigens) expressed by the COVID-19 virus in samples from human respiratory tract. If the targeted antigen is present in sufficient concentration in the sample, it will bind to certain antibodies embedded in a piece of paper embedded in a plastic lump and produce a clear visible signal, usually within 30 min. An example of LFA-based RDT for SARS-CoV-2 is depicted in Figure 6. The antigen(s) found are shown only when the virus is replicated; therefore, such tests are best used to identify severe or early infection. Assay outcome in these assays, however, depends on a number of factors, including the time from the onset of the disease, the viral load in the specimen, the quality of the sample collected from the individual and its processing, and the exact composition of the reagents for test kits. For instance, a handheld POC system which is paired with a smartphone (for result visualization) for rapid detection of SARS-CoV-2 extracted RNA based on reverse-transcription loop-mediated isothermal amplification (RT-LAMP) reported average detection time of 12 min for positive samples [107].

### Neutralizing antibody assay

**Figure 5:**
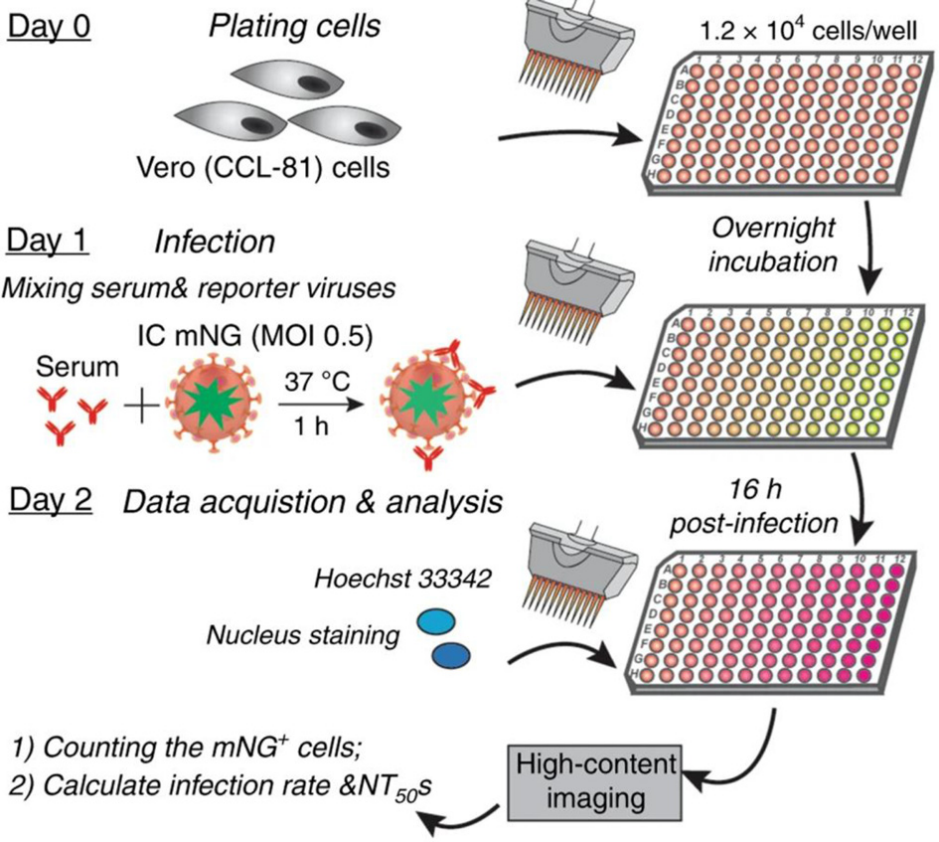
Neutralizing antibody assay Schematic representation of a high-throughput neutralizing antibody assay for COVID-19 diagnosis. Image adapted from [110] with permission from Springer Nature under Creative Commons license.

Infection with SARS-CoV-2 typically induces neutralizing antibody responses [108]. However, only some of these antibodies do have the ability to neutralize the virus particles. A virus neutralization assay is a serological test that detects the presence and magnitude of antibodies that prevent infectivity of the virus and neutralizes the infection [109]. Detecting the neutralizing antibodies help in assessing the ability of existing antibodies to combat the infection and further protect from re-infection of SARS-CoV-2, and identify donors with high-neutralizing titers for convalescent plasma for therapy. The ELISA, LFA, or microsphere immunoassays which measure antibody binding to SARS-CoV-2 spike protein do not functionally measure antibody inhibition of SARS-CoV-2 infection as not all spike-binding antibodies can block viral infection. Therefore, neutralizing antibody assay is considered an ideal serological assay to predict protection from re-infection [110]. Neutralizing antibodies are generally measured by the plaque reduction neutralization test (PRNT). In general, a virus neutralization test involves mixing the patient serum samples with a viral suspension and pouring the resulting solution into a monolayer culture of host cells. If the neutralizing antibodies were present in the patient serum, then the viral replication will be inhibited (neutralized) and there will be no cytopathic effect. In PRNT assay, the cell layer (with added serum-virus suspension) is covered with a layer of agar and the concentration of plaque forming units is estimated by counting by microscope the number of plaques (regions of infected cells) formed after a few days [111]. However, PRNT assay is not suited for large-scale serodiagnosis, and newer methods are required to detect SARS-CoV-2 neutralizing antibodies in patient specimens. A recent publication by Muruato et al. (2020) reported a fluorescence-based high-throughput neutralization assay (Figure 5) for SARS-CoV-2 by infecting Vero CCL-81 cells with the virus/serum mixture of mNeonGreen SARS-CoV-2 (an infectious cDNA clone of SARS-CoV-2) and COVID-19 patient sera, and quantifying the fluorescence of infected cells to estimate the NT_50_ value for each serum [110].

### Biosensors

A biosensor is an analytical tool consists of a biorecognition element to bind the analyte of interest, and a transducer to produce the assay outcomes [112]. Biosensor use has been largely reported in food industry, disease and treatment monitoring, environmental monitoring, and research developments [113]. In past years, development of multiple biosensor-based methods of SARS-CoV-2 detection has been reported (Table 1). Available literature shows development of fluorescence-based, colorimetric, localized surface plasmon resonance (LSPR), surface-enhanced Raman scattering (SERS), quartz crystal microbalance (QCM), field-effect transistor (FET)-based, and electrochemical biosensors for COVID-19 detection [112–114].

**Table 1:** List of various biosensors developed for the detection of Covid-19

Sl. No.	Biosensor type	Sample vol. (µl)	LOD	Sensitivity (%)	Reference
1	Electrochemical	-	15 fM	-	[115]
			90 fM	-	[116]
2	Chip-based	10	30–1000 CFU/ml	-	[117]
3	Paper-based	20	-	91.54	[118]
		10	1 ng/ml	>90	[119]
4	Film-based	500	30 CFU/ml	-	[120]
5	2D material based	5	0.01 CFU/ml	-	[121]
6	Real-time qRT-PCR	5	3.2 copy/µl	-	[122]
7	LAMP	-	-	-	[123]
8	CRISPR-based LAMP with LFA	<10 µl	10 copy/µl	95%	[124]
9	RT-LAMP	25 µl	20 copy/reaction	100%	[125]
10	RCA with MNP	-	Sub-fM	-	[126]
11	Isothermal DNA amplification	-	-	95%	[127]
12	LAMP with colorimetric read out	20 µl	4.8 copy/µl	-	[128]

Abbreviations: CFU, colony forming unit; fM, femtomolar; LOD, limit of detection; RT, reverse transcription.

Examining the interaction between photons and surface electrons at the intersection of nanomaterials has been used to develop plasmonic biosensors. For example, the prism-based surface plasmon resonance (SPR) biosensors have been widely used for real-time chemical and biological applications. Recent adoption of plasmonic fiber-optic biosensor has also overcome the bulkiness issue and added new characteristics like tolerance to harsh environment and remote sensing to plasmonic biosensors [129]. SPR biosensors are also employed to detect the SARS-CoV antibody where a protein synthetic gene was coupled with gold-binding genes of SARS coronaviral surface antigen polypeptides [130]. Recently, a team of Masson investigators reported direct detection of SARS-CoV-2 nucleocapsid antibodies in human serum sample using SPR-based sensors [131]. The peptide monolayer was successfully coated on the metal surface of SPR sensor and allowed to interact with the nucleocapsid protein of the coronavirus that eventually detected SARS-CoV-2 antibodies at the nanomolar concentration. EC sensors that offer greater advantages, such as low cost, high sensitivity, ease of use, and comparatively easy instrumentation are also reported used for the detection of SARS-CoV-2. Recently, molecularly imprinted polymers (MIPs)-based electrochemical sensor has been fabricated by Raziq et al. [115] for the detection of SARS-CoV-2 nucleoprotein antigen with a detection limit ∼15 fM. FET-based biosensing platforms that allows achieving high sensitivity with low volume samples for rapid analyses are another promising tools for clinical and POCT of SARS-CoV-2 [132]. Seo et al. have successfully developed an FET-based tool for detection of SARS-CoV-2 in clinical specimens [133].

**Figure 6:**
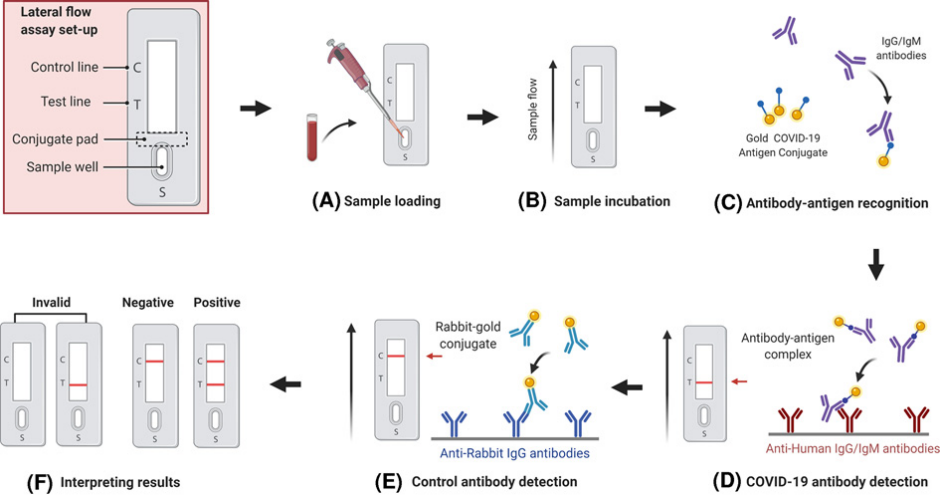
Schematic illustration of an LFA-based RDT biosensor for SARS-CoV-2 antibody (IgM–IgG) detection in blood samples Image shows sample loading, incubation, antigen–antibody reaction, and development of colored product (lines) for easy interpretation of assay result (**A–F**). Incubation allows the capillary action to move sample antibodies (IgG/IgM) forward towards conjugate pad where sample antibodies interact and form complexes with AuNP-coated antigens that are next immobilized by anti-human IgG/IgM antibodies and displayed as test and control red lines. Image adapted from reference [103] with permission from *PeerJ* under Creative Commons Attribution License.

Recently, paper-based biosensors have attracted more attention for POC testing applications in various field of molecular diagnostics due to its low cost, biodegradability, simplicity of design and operation, and high efficiency [134–136]. In particular, paper-based LFAs have been widely used for the detection of COVID-19, specifically IgG and IgM in whole blood, serum, and plasma samples [137,138]. Most of these biosensors are developed as RDTs for the SARS-CoV-2 antigen (Ag-RDT) or antibody (Ab-RDT). Using nasopharyngeal secretions (for Ag-RDT) or finger prick capillary blood specimens (for Ab-RDT), these biosensor-based RDTs allow communities to detect SARS-CoV-2 infection without laboratory infrastructure and trained manpower at their doorstep. Most of the POC biosensor-based RDTs are designed on the principle of LFA wherein a nitrocellulose membrane strip having a sample pad and conjugate pad (gold nanoparticle–antibody conjugates or gold nanoparticle–antigen conjugate) on one end and anti-rabbit IgG (control line) and anti-human IgG/IgM (test lines) on the other [137,138]. The blood or nasopharyngeal secretions added on to the sample pad are drawn through the membrane strip by capillary action, and as it passes the first line/s it produces a visible red color (Figure 6). RDTs are generally rapid assays, but have a low diagnostic performance compared with ELISA and have high rate of false-negativity due to low or variable viral load, as well as sample variability [139].

### Molecular tests (nucleic acid based) for detection of SARS-CoV-2

The availability of the entire genetic sequence of SARS-CoV-2 on global platform (Global Initiative on Sharing All Influenza Data; GISAID) allowed researchers and companies to design primers and probes to develop a range of molecular diagnostic tests for SARS-CoV-2. The key nucleic acid-based tests are RT-PCR, RT-LAMP, transcription-mediated amplification (TMA), CRISPR-based assay, rolling cycle amplification, hybridization-based microarray, and high-throughput sequencing.

### RT-PCR

Polymerase chain reaction (PCR) is a very sensitive and currently the gold standard method for SARS-CoV-2 virus detection. The RT-PCR tests for COVID-19 typically use samples collected from the upper respiratory system using swabs to isolate viral RNA, which is then converted into cDNA, and amplified with specific sets of primers targeting highly preserved and/or highly expressed genes such as the spike glycoprotein gene (S gene), envelope protein gene (E genes) and nucleocapsid protein gene (N genes) [140,141]. Innumerous studies published in last 2 years have discussed in detail the RT-PCR methods/kits for SARS-CoV-2 detection, hence not covered here. Notwithstanding, a few of RT-PCR methods for SARS-CoV-2 detection are listed in (Table 2).

**Table 2:** Representative examples of some RT-PCR testes used in COVID-19 detection

Sl. No.	Test name	Manufacturer	Result time
1	iAMP COVID-19 detection kit	Atila BioSystems, Inc. D11	1.5 h
2	BioFire COVID-19 test	BioFire Defense, LLC	45 min
3	CDC 2019-Novel Coronavirus RT-PCR Diagnostic Panel	CDC-US	-
4	Xpert Xpress SARS-CoV-2 test	Cepheid	45 min
5	VitaPCR SARS-CoV-2 assay	Credo Diagnostics Biomedical Pte Ltd.	20 min
6	LYRA SARS-CoV-2 assay	Diagnostic Hybrids, Inc. Quidel Corporation	75 min
7	SARS-CoV-2 assay	DML-Northwestern Medicine	1 h
8	Simplexa COVID-19 Direct	DiaSorin Molecular LLC	1 h
9	ePlex SARS-CoV-2 test	GenMark Diagnostics, Inc.	2 h
10	Panther Fusion SARS-CoV-2 assay (Panther Fusion System)	Hologic Inc.	3 h
11	COVID-19 RT-PCR test	LabCorp Laboratory Corporation of America	2–4 days
12	ARIES SARS-CoV-2 assay	Luminex Corporation	2 h
13	Accula SARS-CoV-2 test	Mesa Biotech Inc.	30 min
14	MiRXES FORTITUDE KIT 2.0	MiRXES Pte Ltd.	90 min
15	QIAstat-Dx Respiratory SARS-CoV-2 panel	Qiagen GmbH	1 h
16	cobas SARS-CoV-2	Roche Molecular Systems, Inc.	3.5 h
17	TaqPath COVID-19 combo kit	Thermo Fisher-Applied Biosystems	-
18	Novel Coronavirus (2019-nCoV) Nucleic Acid diagnostic kit	Sansure Biotech Inc.	30 min
19	STANDARD M nCoV RT detection kit	SD BIOSENSOR	90 min
20	Allplex 2019-nCoV assay	Seegene	2 h
21	Viracor SARS-CoV-2 assay	Viracor Eurofins Clinical Diagnostics	12–18 h

Table adapted from [142] under an ACS Author Choice License.

### CRISPR diagnostics

**Figure 7:**
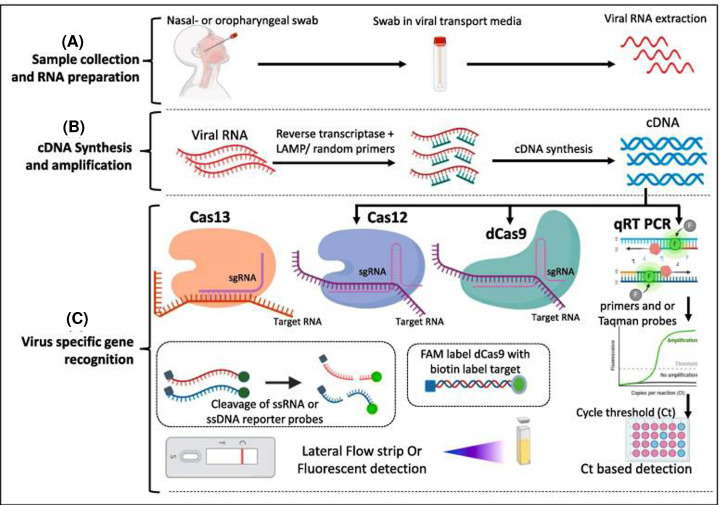
A representative CRISPR-based method for COVID-19 detection The method comprises sample collection and RNA isolation (**A**), conversion of RNA into cDNA through recombinase polymerase amplification (**B**), and virus-specific gene detection through conventional qRT-PCR or advanced Cas-based enzymes (**C**). Image adapted from [145] with permission from Elsevier under COVID-19 resource center.

The CRISPR-based lateral-flow assays are a new addition to the RDT. Going beyond its ability to function as ‘cellular scissors’, CRISPR and its related proteins indicate structures that can be used to obtain specific nucleic acids in a sample [143–144]. The method can detect RNA or DNA of viruses through reverse transcription reaction with detection of positive samples using fluorescent reporter and quencher couple (Figure 7). Some of the notable example of CRISPR-based tests for COVID-19 are SHERLOCK, AIOD-CRISPR, DETECTR, ENHANCE, and FELUDA [23]. CRISPR diagnostic methods benefit from high molecular sensitivity and selectivity, and rapid activation and facile use for lateral-flow assays testing. They are able to adapt to new goals and provide readings that can be faster and clearer.

## Conclusions and perspectives

In the past two decades, humans have witnessed the pandemics of severe acute respiratory syndrome (SARS), swine flu, ebola virus disease (EVD), middle east respiratory syndrome (MERS), and COVID-19. The COVID-19 is a life-threatening respiratory disease caused by SARS-CoV-2, a novel and the third most deadly human coronavirus. Upon its first appearance in December 2019 in Wuhan, China, the disease rapidly spread to 90% countries in just 2 months, and declared a pandemic by the WHO in March 2020. It has infected more than 160 million people, and accounted for over 3.3 million deaths globally so far until 13 May 2021. The virus infects mainly the lung epithelial cells via membrane fusion of its surface protein S1 with human receptor ACE2 present over lung, nose, and mouth epithelial cells. Having greater human transmission capacity than other human coronaviruses, SARS-CoV-2 infection continues to surge in multiple waves in different countries. The urgent need for accurate and rapid diagnosis of SARS-CoV-2 infection remains crucial as global healthcare systems continue to curtail its spread with limited vaccines and no therapeutic drugs. This review provides an overview of the structural makeup and transmission modes of the virus and discusses various methods reported for rapid and accurate detection of SARS-CoV-2 over the past decade. Besides the most widely used qRT-PCR, we provided a detailed insight into various serological and immunological tests such as ELISA, LFA, neutralizing antibody assay, rapid antigen test, and biosensor as well as various molecular (nucleic acid-based) tests such as CRISPR diagnostics. While multiple reports on efficient and cost-effective methods and tools for SARS-CoV-2 testing keep developing (Table 3), qRT-PCR that detect SARS-CoV-2 based on its unique genetic makeup remained the most widely used testing method for COVID-19. Amidst continuous surge in SARS-CoV-2 infection all across the globe, molecular tests such the CRISPR-based diagnostic systems could provide COVID-19 detection methods with added advantages of detection speed (e.g., 30 min from sampling to result), high sensitivity and precision, portability, and no need for specialized laboratory equipment. Serological and immunological tests like ELISA, LFA, and neutralizing antibody assays could help in determining the extent of population-based spread of infection (e.g., India’s Sero-Surveillance), as well as to predict protection from re-infection and identify donors with high-neutralizing titers for convalescent plasma for therapy. Use of biosensors and nanoscale visualization or characterization tools can be another promising strategy for timely/real-time diagnosis of COVID-19, and to decrease COVID-19 causalities arising from time lapse between infection and detection. Yet, the constant mutation in virus that enables it to alter genetic makeup as well as surface expressed proteins, making it hard for already available diagnostics to detect it with high precision needs to be addressed comprehensively.

**Table 3:** List of various COVID-19 tests with manufacturer name, test type, price (INR), specificity, and sensitivity

Sl. No.	Name of the test	Manufacturer	Type of the test	Price (INR)	Specificity (%)	Sensitivity (%)
1	LAMP Covid-19 detection kit	Atila Biosystems	Real-time RT isothermal amplification test	200	97.6	97.60
2	CRISPR- based tests for Covid-19	Cepheid Sherlock Biosciences, Mammoth Biosciences	CRISPR-based LFA isothermal amplification	500	100	90
3	Xpert Xpress SARS-CoV-2	Cepheid	Real- time PCR	600–700	100	71
4	VITROS-Immunodiagnostics Products Anti-SARS-CoV total reagent pack	Ortho-clinical diagnostics	ELISA	300	100	87.30
5	Onsite Covid-19 IgG/IgM rapid test	CTK Biotech Inc. (U.S.A.)	Lateral flow immunoassay	200	99.00	99.00
6	MAGLUMI IgG/IgM de 2019-nCoV (CLIA)	Snibe Diagnostic	Chemiluminescence immunoassay	350	99.20	86.90
7	M2000 SARS-CoV assay	Abbott Core Laboratory	Chemiluminescent microparticle immunoassay	400	100	93
8	ID NOW COVID-19	Abbott diagnostics Scarborough, Inc.	Isothermal nucleic acid amplification	100–200	95	95
9	One-step COVID-2019 test	Celer Biotechnologies	Lateral flow immunoassay	100	99.57	86.43
10	Cobas HBeAG detection kit	Roche Diagnostics, India	Electrochemical-luminescence immunoassay	500–600	99.20	100
11	DSI Covid-19 Ag Rapid test kit	IIT Delhi, India	Immunochromatography	50	90	100
12	Coviself Covid-19 Rapid Ag Test Self Test Kit	Mylab, India	Immunoassay	250	-	-
13	Covid-19 Ag Rapid Test Device home test kit	Abbott, India	Immunoassay	325	-	-

While we have no doubt that the COVID-19 pandemic will be successfully fought in the coming future, the lessons we should learn about the alarming number of deaths and the great economic crisis involved should inform us of the need to properly prepare for any outbreak. Also, while the past few months have seen rapid progress in the development of COVID-19 diagnostic kits, the race continues to develop more effective laboratory techniques and cost-efficient, diagnostic testing centers that can be used in bulk. Ultra-rapid test kits and POCT focus on development to accelerate treatment response time and eliminate the need for extended laboratory equipment and waiting time involved with testing in accredited laboratories. To promote more accurate and faster diagnostic solutions, many organizations support these activities by inviting trial developers to present their test products for independent testing or by providing large investments for greater collaboration. As similar programs and information sharing become accessible, including mutual scientific advances, it is likely that the COVID-19 recognition market will continue to grow well in the future.

## Abbreviations

ACE2: angiotensin-converting enzyme 2
COVID-19: coronavirus disease 2019
ELISA: enzyme-linked immunosorbent assay
FET: field-effect transistor
IgG: immunoglobulin G
IgM: immunoglobulin M
LAMP: loop-mediated isothermal amplification
LFA: lateral flow assay
MERS-CoV: Middle East respiratory syndrome virus
POCT: point-of-care testing
PRNT: plaque reduction neutralization test
RDT: rapid diagnostic test
RT-LAMP: reverse-transcription loop-mediated isothermal amplification
RT-PCR: reverse-transcription polymerase chain reaction
SARS-CoV: severe acute respiratory syndrome coronavirus
SPR: surface plasmon resonance
TMPRSS2: transmembrane protease serine 2
